# Psychological distress and coping strategies among caregivers of children with cancer: a cross-sectional study

**DOI:** 10.3332/ecancer.2025.1872

**Published:** 2025-03-13

**Authors:** Poonam Joshi, Gopichandran L, Uma Shanker Agrawal, Parveen, Rakesh Garg, Surya Kant Tiwari

**Affiliations:** 1 College of Nursing, All India Institute of Medical Sciences, Kalyani 741245, West Bengal, India; 2 Department of Nursing, National Institute of Mental Health and Neurosciences, Bangaluru 560029, Karnataka, India; 3 BRAIRCH, All India Institute of Medical Sciences, New Delhi 110029, India; 4 Department of Anesthesiology, BRAIRCH, All India Institute of Medical Sciences, New Delhi 110029, India; 5 College of Nursing, All India Institute of Medical Sciences, Raebareli 229405, Uttar Pradesh, India; a https://orcid.org/0000-0002-7016-8437; b https://orcid.org/0000-0003-4718-0398; c https://orcid.org/0000-0002-9855-0742

**Keywords:** adaptation, psychological, anxiety, caregivers, coping skills, depression, mental health

## Abstract

**Background:**

Caregivers of children with cancer often experience significant psychological distress exacerbated by financial constraints and role modifications. This study aimed to assess the prevalence of anxiety and depression among caregivers of children receiving cancer treatment and to examine their coping mechanisms.

**Methods:**

This cross-sectional study included 124 caregivers of children receiving chemotherapy at a tertiary care facility in Northern India. Inclusion criteria included caregivers more than 18 years of age, engaged in child care for at least 3 months, accompanying them for chemotherapy and understanding Hindi or English. Anxiety and depression were assessed using the Hospital Anxiety and Depression Scale. The coping strategies were evaluated using a Brief Coping Orientation to the Problem-Experienced inventory.

**Results:**

The mean age of the caregivers was 34.50 ± 7.60 years, with the majority being male (62.1%). Over one-third of the caregivers (37.9%) reported abnormal anxiety levels, while 44.4% exhibited borderline depression. A significant moderate positive correlation was found between anxiety and depression (*r* = 0.676, *p* < 0.001). The most commonly used coping mechanisms are active coping and self-distraction. Multiple regression analysis revealed self-blame, acceptance and planning as significant coping strategies for anxiety, while self-blame, planning and religion were significant for depression.

**Conclusion:**

A substantial proportion of the caregivers experienced anxiety and depression. Emotion-focused and problem-focused coping strategies were the most frequently employed. These findings highlight the need for psychosocial support and interventions to foster healthier coping skills among caregivers of children with cancer.

## Key-points

- Caregivers of children with cancer face significant psychological distress, with over one-third experiencing anxiety and approximately half exhibiting borderline depression.
- The coping mechanisms utilised by caregivers include active coping, self-distraction, acceptance, planning and religion, with self-blame being a common coping strategy for both anxiety and depression.
- There was a significant moderate positive correlation between anxiety and depression among caregivers, highlighting the interconnectedness of these psychological issues.
- Healthcare professionals should implement tailored counseling services, psychosocial support and coping strategy training to alleviate psychological distress and enhance the overall well-being of caregivers in pediatric cancer care settings.

## Background

Cancer is the leading contributor to global mortality, accounting for approximately 19.3 million cases and approximately 10 million deaths in 2020 [1, 2]. India is ranked third, trailing behind China and the United States of America. The Indian National Cancer Registry Program has an estimated crude incidence rate of 100.4/100,000 for the year 2022 [3]. Lymphoid leukemia and neuroblastoma are the most frequently diagnosed childhood cancers in both sexes of India.

Coping with cancer among children not only poses a significant emotional burden on the child, but also profoundly affects caregivers. Despite advancements in diagnosis and treatment, and improvements in survival rates, the diagnosis of cancer is extremely difficult for parents [4]. This impact extends beyond social and cultural factors within the Indian population and covers additional challenges related to financial and psychological aspects. Given the heightened dependence of younger children on their caregivers to fulfill various needs, caregivers’ concerns are multifaceted. They grapple with anxieties related to their children’s education, development and future societal roles in addition to addressing the physical and psychological symptoms expressed by the children [5].

The psychological distress faced by caregivers often manifests as anxiety and depression, arising from concerns about the potential outcomes, morbidity and mortality of the affected child with cancer [6–8]. Financial constraints exaggerate this situation. Caregivers also modify their roles, responsibilities and family functions to accommodate their children [8]. Such concerns contribute to psychological distress among caregivers, requiring a unique approach to conceptualise the problems they face [9, 10].

A recent systematic review and meta-analysis revealed a prevalence of depression of 42.5% and anxiety of 46.55% among caregivers of patients with cancer [11]. Another meta-analysis revealed a higher rate of depression among female caregivers [12]. The psychological issues among caregivers have gone unnoticed despite the higher prevalence of mental health issues and, consequently, have not been adequately addressed in clinical settings.

Pediatric cancer care poses unique challenges. While previous studies have documented the psychological burden on caregivers, there is a dearth of research focusing on the specific coping mechanisms employed by caregivers of children with cancer in India. This gap hinders the development of targeted interventions to support this vulnerable population. This study sought to bridge these gaps by providing empirical evidence on the prevalence of psychological distress and the efficacy of coping strategies among caregivers of children with cancer in India. By identifying the most prevalent coping mechanisms and their impact on caregivers’ mental health, our research aimed to inform the development of targeted psychosocial interventions tailored to the cultural and contextual realities of the Indian caregiving environment.

## Methods

### Study design and setting

This descriptive cross-sectional study was conducted among caregivers of children with cancer attending the daycare center (DCC) for chemotherapy at the pediatric oncology unit of a tertiary care facility in Northern India from August to October 2021. The participants were selected using a convenience sampling technique because of the study’s specific focus on caregivers of children with cancer at a chemotherapy DCC and logistical constraints. This approach allowed for efficient data collection within the study timeframe, as the center provided direct access to the target population, overcoming time and resource limitations that precluded a more comprehensive sampling strategy. The study was conducted and reported in accordance with the Strengthening the Reporting of Observational Studies in Epidemiology guidelines [13].

### Target population

The target population in this study was the primary caregivers. In this research, the term ‘caregivers’ was specifically defined as mothers, fathers or any key family member who directly provided care or held primary responsibility for supporting and caring for children diagnosed with cancer. All caregivers who were more than 18 years old, involved in the child’s care for at least 3 months, accompanied by chemotherapy, willing to participate or capable of understanding the Hindi or English language were included.

### Ethical considerations

The research was conducted with approval from the Institutional Ethics Committee (IEC-762/2020) and in accordance with the 2013 revision of the 1975 Helsinki Declaration. Each participant received a thorough explanation of the study objectives before providing written informed consent. Involvement in the study was optional, and the researcher assured the participants that their information would be kept confidential and anonymous.

### Sample size

Based on a previous study reporting caregiver anxiety and depression incidence rates of 41.2% and 32.4%, respectively [5], we calculated a sample size of 124. Using a 5% α-level and 10% absolute allowable error in the estimation of the prevalence of anxiety and depression, sample sizes of 94 and 85 were calculated, respectively. The sample size was adjusted to achieve an 80% response rate.

### Tools for data collection

A validated three-part questionnaire was used for the data collection. The first tool gathered sociodemographic information about caregivers and their children, including details such as age, sex, marital status, family type, residence and employment status. The children also collected data on age, sex, education, diagnosis, chemotherapy duration and number of treatment cycles.

The second tool used the Hospital Anxiety and Depression Scale (HADS) [14], a standardised 14-item measure assessing anxiety and depression. Each subscale contains seven items scored on a four-point Likert Scale (0 = not at all; 3 = most of the time), with a maximum possible score of 21 for both anxiety and depression. The scores were classified as normal (0–7), mild (8–10), moderate (11–14) or severe (15–21). The HADS demonstrated acceptable reliability in this study, with a Cronbach’s alpha value of 0.757.

The third tool was the 28-item self-report Brief Coping Orientation to the Problem-Experienced (COPE) inventory [15]. Coping strategies were evaluated using a four-point Likert scale ranging from 1 (I have not been doing this at all) to 4 (I have been doing this a lot), with higher scores indicating more frequent use of a particular coping mechanism. The Brief COPE assesses 14 coping mechanisms, each consisting of two items, and examines three primary coping strategies: emotion-focused, problem-focused and avoidant coping.

Problem-focused coping includes active coping, use of informational support, planning and positive reframing. Higher scores in this category indicate strategies aimed at altering stressful situations. Emotion-focused coping includes venting, use of emotional support, humour, acceptance, self-blame and religion. Higher scores in this area suggest strategies focused on managing the emotions associated with stressful situations. Avoidant coping involves self-distraction, denial, substance use and behavioural disengagement, with higher scores indicating an effort to physically or cognitively distance oneself from stressors. The Brief COPE exhibited acceptable reliability, with a Cronbach’s alpha of 0.793.

### Procedure for data collection

To ensure accuracy, the questionnaires were translated into Hindi and back-translated with validation from the Institute’s Hindi Department. The researchers established a rapport with caregivers of pediatric cancer patients who met the inclusion criteria. Participants were given a thorough explanation of the study, including an information sheet and written informed consent was obtained, guaranteeing the confidentiality and anonymity of the collected data. Eligible caregivers were provided with all necessary tools for data collection when their children were admitted to the DCC for chemotherapy. Clear guidance was given on how to complete the questionnaire, which took approximately 10–15 minutes. Following the survey, the researchers addressed questions from caregivers and expressed their gratitude for their participation and time.

### Data analysis

Microsoft Excel was utilised for data coding and summarisation. IBM SPSS Statistics for Windows, Version 26.0 (IBM Corp., Armonk, NY) was employed for both descriptive and inferential statistical analyses. Descriptive statistics included frequency, percentage, mean, standard deviation, median and interquartile range. The internal consistency of the scale was evaluated using Cronbach’s alpha. An independent samples *t*-test was conducted to determine the mean differences among the study variables. Pearson’s correlation coefficient (*r*) was calculated to assess the relationship between anxiety, depression and coping strategies. The association between psychological distress and coping strategies was examined using multiple linear regression analysis. Statistical significance was set at *p* < 0.05.

## Results

Most caregivers were males (62.1%), had a mean age of 34.50 ± 7.60 years, were married (89.5%), and resided in an urban area (66.9%). The mean age of the children was 9.21 ± 4.68 years, with the majority being males (64.5%) and having solid tumours (57.2%). The median duration of chemotherapy during admission to daycare was 3 (3–4) hours (Table 1).

More than one-third of the participants experienced abnormal anxiety (37.9%) and approximately half of the caregivers exhibited borderline depression (44.4%). A statistically significant moderate positive correlation was observed between anxiety and depression (*r* = 0.676, *p* < 0.001). Moderate positive correlations were found between anxiety and self-blame (*r* = 0.460, *p* < 0.001) and between depression and self-blame (*r* = 0.465, *p* ≤ 0.001). These findings suggest that maladaptive coping mechanisms are associated with both anxiety and depression (Table 2).

Table 3 describes the differences in mean scores between coping mechanisms, anxiety and depression. Problem-focused coping mechanisms, such as planning, positive reframing and the use of informational support, are frequently employed by both non-anxious and non-depressed groups. Similarly, emotion-focused coping mechanisms such as acceptance, emotional support and religion were common among both groups. Furthermore, significant differences in mean scores were observed for emotion- and problem-focused coping mechanisms in both the anxious and depressed groups.

Multiple linear regression analysis was conducted to examine the effect of coping strategies (only significant variables) on anxiety (Model 1) and depression (Model 2). Both models were found to be significant, indicating that the current multiple regression model was a good fit for the data: Model 1 (*F* = 10.580, *p* < 0.001). Adj *R*^2^ = 0.353) and Model 2 (*F* = 10.367, *p* < 0.001, Adj *R*^2^ = 0.407). The multiple correlation coefficient (*R*) values were 0.624 and 0.671, demonstrating a moderate relationship between coping mechanisms and anxiety and depression. The coefficient of determination (*R*^2^) values of 0.390 and 0.450 suggest that coping mechanisms explained 39.0% and 45.0% of the variance in anxiety and depression, respectively.

In Model 1, self-blame (*β* = 0.344, *p* < 0.001), acceptance (*β* = −0.244, *p* < 0.001) and planning (*β* = −0.231, *p* = 0.016) were identified as significant and effective coping strategies for dealing with anxiety symptoms. In Model 2, self-blame (*β* = 0.227, *p* = 0.013), planning (*β* = −0.198, *p* = 0.040) and religion (*β* = −0.189, *p* = 0.047) emerged as significant effective coping strategies for managing depressive symptoms (Table 4).

## Discussion

This study aimed to assess the prevalence and severity of psychological distress among caregivers of children with cancer who were receiving chemotherapy. The major findings of our study suggested that anxiety was common in more than one-third of the participants, while borderline depression was evident in approximately half of the caregivers. A statistically significant moderate positive correlation was observed between anxiety and depression. A multiple linear regression analysis revealed significant coping strategies for anxiety (self-blame, acceptance and planning) and depression (self-blame, planning and religion).

Screening for psychological problems is a critical element of comprehensive psychosocial care for cancer patients and their families [16]. In this study, we used the HADS to assess psychological problems among caregivers of children with cancer. A key finding was that approximately one-third of the caregivers had abnormal anxiety and depression, which is consistent with the findings of various studies [17, 18]. A recent study from Iran demonstrated that approximately half of caregivers of patients with cancer had severe depression and anxiety, which significantly predicted caregiver burden [19]. Another study reported that depressive symptoms were common in informal caregivers from the COVID-19 Baseline to Exit survey, while anxiety was not as prevalent [20]. In contrast to the present findings, a study from China showed that depression, anxiety and stress among cancer caregivers were within the normal ranges [21].

Abnormal levels of anxiety and depression among caregivers can be attributed to several factors. Caregivers have to face the painful realities of a cancer diagnosis. A qualitative investigation revealed that the primary concern among caregivers was the gradual decline in their loved one’s health and awareness of their nearing death [22]. They are also susceptible to experiencing significant psychological distress from the time of cancer diagnosis, with extreme depressive symptoms remaining prevalent in about one-quarter of caregivers [23]. Furthermore, a recent assessment of childhood cancer care services in India highlighted the concentration of childhood cancer care services available at the tertiary level of health care, emphasising gaps in specialised pediatric oncology care across tertiary hospitals [24]. Previous studies have indicated that variables such as younger age, female sex, primary cancer and past surgery are associated with caregivers’ depression and anxiety [25–27]. Additionally, financial and treatment burdens might also increase depression and anxiety levels among caregivers, as the majority of participants in our study reported not receiving financial support from any government, non-governmental organisation or insurance agency.

Dealing with illnesses such as cancer in children is consistently challenging for family members. Another interesting finding of our study was that the most commonly used coping mechanism among caregivers was emotion-focused coping, followed by problem-focused coping, consistent with previous studies [28–31]. The least-utilised coping mechanism in our study was avoidant coping. Active coping was the most frequently used individual coping strategy, while substance abuse was the least common among caregivers. A Sri Lankan study showed that seeking medical help through communication with parents and consultation with medical staff was the least common maternal coping strategy in response to a child’s oncological disease [32]. A previous study showed that caregivers of children with physical and mental disabilities employed active emotional coping strategies more frequently [33]. Few qualitative studies have reported coping strategy themes among cancer caregivers as physical activity, family cohesiveness, spirituality, hobbies, community and social support and avoidance [34, 35].

The moderate positive correlation between anxiety and depression in our study highlights the complex interplay between these two mental health conditions. Several factors may contribute to this relationship including shared risk factors, symptom overlap and comorbidities [36]. Common environmental stressors or genetic predispositions can simultaneously influence anxiety and depression [37]. Additionally, the overlap in symptoms, such as sleep disturbances or difficulty in concentrating, may partially account for the observed correlation [38, 39]. The frequent co-occurrence of anxiety and depression in clinical settings further supports this relationship [40]. It is also important to consider the potential temporal relationship between these conditions, as prolonged anxiety may lead to depressive symptoms or vice versa [41, 42].

Moderate negative correlations were found between anxiety and acceptance, planning and religion. Similarly, moderate negative correlations were observed between depression and acceptance, planning, positive reframing, emotional support and religion. A recent study revealed that caregivers’ stress levels were positively correlated with active coping, denial, behavioural disengagement, humour, acceptance, religion and self-blame [43].

Multiple linear regression analysis showed that planning and self-blame were effective coping strategies for both depressive and anxiety symptoms. This shows that caregivers who prepared their minds coped more effectively than those who did not. Caregivers may also blame themselves for their loved one’s illness, whether due to their perceived inability to provide sufficient care or to prevent it, which can lead to feelings of guilt, sadness and hopelessness. Another intriguing finding of our study is that acceptance and religion were significant coping strategies for anxiety and depression. Engaging in religious or spiritual practices can serve as an adaptive resource, offering comfort and support to caregivers in dealing with depressive symptoms.

### Strengths of the study

This study employed a comprehensive methodology with an adequate sample size to explore psychological distress and coping mechanisms among caregivers of children undergoing chemotherapy. Furthermore, we used standardised and validated instruments to enhance the reliability and validity of our findings.

## Limitations

Several constraints should be considered when evaluating the study outcomes. First, the cross-sectional nature of the study restricted our capacity to establish causal relationships or to fully comprehend the temporal interactions between anxiety, depression and coping strategies. Second, the use of self-administered questionnaires may have introduced a response bias. Third, the convenience sampling approach and single-site setting may limit the applicability of our results to a wider population. Furthermore, although our statistical model accounted for a substantial portion of the variance in anxiety (39%) and depression (45%), a significant amount remained unexplained, indicating that additional factors not examined in this study may have influenced these multifaceted phenomena. To address these limitations, future investigations should employ multicenter longitudinal designs with larger participant pools and explore other variables that may impact anxiety and depression.

### Clinical implications

Based on the findings of this study, several interventions can be implemented in hospitals to support caregivers of pediatric cancer patients. Tailored coping strategy training can be developed, focusing on culturally sensitive workshops that emphasise active coping, self-distraction, acceptance and planning. These workshops should incorporate religious coping techniques, respect the cultural context in India, and provide practical exercises to enhance these skills. Comprehensive mental health screening should be implemented, including routine anxiety and depression screening for caregivers at regular intervals, using culturally validated tools specific to the Indian context. Clear referral pathways should be established for caregivers who screen positively for psychological distress. Psychoeducation programs should be conducted to educate caregivers about the potential psychological impacts of caregiving, recognising signs of anxiety and depression, and providing guidance on seeking professional help. Peer support groups should be facilitated to allow caregivers to share experiences and coping strategies with separate groups for different treatment stages. Culturally adapted mindfulness-based stress reduction programs can be introduced to help reduce anxiety and depression among caregivers [44].

## Conclusion

In conclusion, this study revealed significant psychological distress among caregivers, with over one-third experiencing abnormal anxiety levels and nearly half experiencing borderline depression. The most prevalent coping strategies were active coping and self-distraction. A moderate positive correlation was found between anxiety and depressive symptoms. Notably, specific coping mechanisms were associated with different psychological outcomes: self-blame, acceptance and planning were significantly linked to anxiety, whereas self-blame, planning and religion were significantly associated with depression. These findings underscore the complex interplay among caregiving stress, psychological well-being and coping strategies, highlighting the need for targeted interventions to support caregivers’ mental health.

## Conflicts of interest

The authors (s) declare that they have no conflicts of interest.

## Funding

This research did not receive any specific grants from funding agencies in the public, commercial or not-for-profit sectors.

## Ethical statement

The Institutional Ethics Committee of the All India Institute of Medical Sciences, New Delhi, reviewed and approved the study vide IEC-762/2020.

## Figures and Tables

**Table 1.** Socio-demographic profile related to caregivers and the child (*n* = 124).

Variables	Frequency (%)
**Related to caregivers**	
Age (years)*	34.50 ± 7.60
Sex Female Male	47 (37.9) 77 (62.1)
Education Primary Middle High school Secondary Graduation and above	23 (18.5) 26 (21.0) 26 (21.0) 19 (15.3) 30 (24.2)
Marital status Married Widow/Divorced	111 (89.5) 13 (10.5)
Type of family Single Joint	60 (48.4) 64 (51.6)
Residence Rural Urban	41 (33.1) 83 (66.9)
Employment status Govt Job Private Job Unemployed Housewife Business Farmer	27 (21.8) 14 (11.3) 30 (24.2) 35 (28.2) 13 (10.5) 5 (4.0)
Treatment burden Government Insurance Self Non-Governemental Organizations	20 (16.1) 2 (1.6) 94 (75.8) 8 (6.5)
Family Income (monthly)**	10,000 (7,000–20,000)
Number of family members**	5 (4–8)
Duration as a caregiver (years)**	6 (3–12)
Related to child	
Age (years)*	9.21 ± 4.68
Sexç Female Male	44 (35.5) 80 (64.5)
Education Not started Primary Middle Secondary	39 (31.5) 46 (37.1) 21 (16.9) 18 (14.5)
Diagnosis Solid tumor Liquid tumor	71 (57.2) 53 (42.8)
Duration of chemotherapy (hours)**	3 (3–4)
Number of chemotherapy cycles**	3 (2–6)

* mean ± SD,

** median(IQR)

**Table 2.** Correlations, descriptives and frequencies of study variables.

	Anxiety (*r*)	Depression (r)
**Anxiety**	1	0.676**
**Depression**	0.676**	1
Self-blame	0.460**	0.465**
Venting	−0.018	−0.020
Behavioral disengagement	0.084	0.108
Substance use	−0.101	0.042
Denial	−0.111	−0.126
Self-distraction	0.086	0.169
Humor	−0.069	−0.093
Acceptance	−0.450**	−0.434**
Planning	−0.409**	−0.448**
Positive reframing	−0.350**	−0.419**
Use of informational support	−0.189*	−0.386**
Emotional support	−0.321**	−0.461**
Active coping	−0.137	−0.179*
Religion	−0.419**	−0.499**
Mean (SD)	9.34 ± 4.73	9.19 ± 3.83
Normal (%)	41 (33.1)	31 (25.0)
Borderline (%)	36 (29.0)	55 (44.4)
Abnormal (%)	47 (37.9)	38 (30.6)

*r* = correlation coefficient; * statistically significant where *p* < 0.05 and two tailed; ** statistically significant where *p* < 0.01 and two tailed

**Table 3.** Coping strategies adopted by cancer caregivers in comparison with study variables (*n* = 124).

Coping mechanisms	Mean (SD)						
	**Total**	**Anxious group**	**Non-anxious group**	***p*-value**	**Depressed group**	**Non-depressed group**	***p*-value**
Self-blame	4.95 (1.71)	6.21 (1.25)	4.18 (1.43)	<0.001**	6.61 (1.12)	4.22 (1.39)	<0.001**
Venting	4.33 (1.37)	4.32 (1.27)	4.31 (1.44)	0.977	4.45 (1.28)	4.26 (1.41)	0.477
Emotional support	4.35 (1.50)	3.79 (1.33)	4.69 (1.51)	0.001*	3.47 (1.22)	4.73 (1.46)	<0.001**
Humor	3.03 (1.30)	2.81 (0.99)	3.17 (1.44)	0.135	2.74 (0.82)	3.16 (1.44)	0.093
Acceptance	4.70 (1.36)	3.77 (1.08)	5.77 (1.61)	<0.001**	3.74 (1.20)	5.13 (1.58)	<0.001**
Religion	4.43 (1.60)	3.60 (1.07)	4.94 (1.67)	<0.001**	3.24 (0.91)	4.95 (1.57)	<0.001**
Emotion-focused coping	25.77 (4.79)	22.49 (3.17)	26.56 (5.43)	0.019*	24.24 (2.84)	26.45 (5.31)	0.017*
Active coping	5.34 (1.55)	5.06 (1.30)	5.51 (1.66)	0.124	4.97 (1.15)	5.50 (1.67)	0.081
Use of informational support	4.49 (1.54)	4.13 (1.42)	4.71 (1.58)	0.040*	3.58 (1.08)	4.90 (1.54)	<0.001**
Planning	4.10 (1.36)	3.45 (0.97)	4.51 (1.42)	<0.001**	3.24 (0.75)	4.49 (1.40)	<0.001**
Positive reframing	4.27 (1.55)	3.51 (1.04)	4.74 (1.64)	<0.001**	3.34 (0.99)	4.69 (1.58)	<0.001**
Problem-focused coping	18.21 (4.43)	16.15 (3.33)	19.47 (4.57)	<0.001**	15.13 (2.64)	19.57 (4.39)	<0.001**
Self-distraction	4.90 (1.42)	5.15 (1.14)	4.75 (1.55)	0.133	5.42 (0.91)	4.67 (1.54)	0.007*
Denial	4.31 (1.46)	4.23 (1.43)	4.35 (1.49)	0.669	4.29 (1.46)	4.31 (1.47)	0.932
Substance use	2.86 (1.27)	2.74 (1.22)	2.94 (1.31)	0.423	2.82 (1.20)	2.88 (1.31)	0.786
Behavioral disengagement	4.49 (1.15)	4.72 (1.09)	4.35 (1.16)	0.080	4.84 (1.10)	4.34 (1.14)	0.024*
Avoidant coping	16.56 (3.10)	16.85 (2.61)	16.39 (3.38)	0.425	17.37 (2.28)	16.21 (3.36)	0.055

* Statistically significant where *p* < 0.05 and two-tailed;

** statistically significant where *p* < 0.01 and two-tailed

**Table 4.** Multiple linear regression analysis exploring the correlates of anxiety and depression.

	*B*	Standard Error	*β*	*t*	*p*
**Anxiety**					
Constant	10.763	2.074	-	5.189	<0.001**
Self-blame	0.949	0.223	0.344	4.250	<0.001**
Acceptance	−0.718	0.294	−0.244	−2.438	0.016*
Planning	−0.801	0.343	−0.231	−2.332	0.021*
Positive reframing	0.128	0.292	0.042	0.438	0.662
Use of informational support	0.507	0.291	0.166	1.742	0.084
Emotional support	−0.016	0.308	−0.005	−0.051	0.959
Religion	−0.502	0.286	−0.171	−1.752	0.082
**Depression**					
Constant	11.720	1.738	-	6.743	<0.001**
Self-blame	0.508	0.201	0.227	2.529	0.013*
Behavioral disengagement	0.144	0.252	0.043	0.571	0.569
Self-distraction	0.399	0.217	0.148	1.842	0.068
Acceptance	−0.109	0.230	−0.046	−0.472	0.638
Planning	−0.555	0.267	−0.198	−2.081	0.040*
Positive reframing	−0.117	0.229	−0.047	−0.511	0.610
Use of informational support	−0.207	0.227	−0.083	−0.912	0.364
Emotional support	−0.333	0.242	−0.131	−1.373	0.172
Religion	−0.449	0.224	−0.189	−2.005	0.047*

* statistically significant where *p* < 0.05 and two tailed;

** statistically significant where *p* < 0.01 and two tailed
